# Adherence to hydroxyurea, health-related quality of life domains and attitudes towards a smartphone app among Irish adolescents and young adults with sickle cell disease

**DOI:** 10.1007/s11845-021-02588-1

**Published:** 2021-03-20

**Authors:** Helen Fogarty, Alan Gaul, Saifullah Syed, Natalija Aleksejenko, Rosena Geoghegan, Helena Conroy, Edel Crampton, Noel Ngwenya, Emma Tuohy, Corrina McMahon

**Affiliations:** 1 Irish Centre for Vascular Biology, Royal College of Surgeons in Ireland (RCSI), 123 St Stephen’s Green, Dublin, Ireland; 2 Haematology Department, Children’s Health Ireland (CHI) at Crumlin, Dublin, Ireland; 3 Haematology Department, St James’s Hospital, Dublin, Ireland

**Keywords:** Adolescents and young adults (AYA), Health-related quality of life, Hydroxyurea, Medication adherence, Sickle cell disease, Smartphone application

## Abstract

**Introduction:**

SCD patients experience declines in health-related quality of life (HRQOL) domains compared with healthy controls. Despite evidence supporting the benefits of hydroxyurea, medication non-adherence remains problematic, especially in adolescents and young adults (AYA). Adherence barriers include forgetfulness and lack of knowledge. Recently, increased interest in technology-based strategies to improve medication adherence has emerged. No data currently exists on hydroxyurea adherence, HRQOL or perceptions of technology-based tools in the Irish SCD population.

**Methods:**

In order to interrogate these domains among Irish AYA SCD patients we administered an anonymous survey at two tertiary referral centres in Dublin, Ireland, in July 2019.

**Results:**

Sixty-three patients participated; 63% female and 37% male, with a median and mean age of 17 and 19 years, respectively. Average monthly adherence was 76% using a visual analogue scale. Recall barriers were present in 62% while 26% omit hydroxyurea for reasons other than forgetting. Reviewing HRQOL; only 36.5% felt always physically able to engage in recreational activities, while 51% experienced disruption to school/college/work due to pain. Eighty-one percent reported that anxiety about health interferes with their lives and non-adherence correlated with worse HRQOL outcomes. Interest in a smartphone app was expressed by the majority, with daily medication reminders being the most popular feature. Sharing adherence data with doctors and discussion forums were less appealing.

**Conclusions:**

Representing over 10% of the Irish SCD population, our survey provides novel and valuable insights into medication adherence and HRQOL domains. Preferred app features may inform future technology-based interventions to improve medication adherence in SCD and other chronic health conditions.

## Introduction

Sickle cell disease (SCD) is a group of inherited haemoglobin disorders, caused by the sickle beta globin gene mutation and is associated with significant morbidity and mortality including chronic haemolysis, stroke and long-term end-organ damage [1]. Compared with healthy controls, SCD patients experience significant declines in health-related quality of life (HRQOL) outcomes over time [2–4]. Globally, SCD affects over 300,000 births per year [5, 6], a figure projected to rise to over 400,000 by 2050 [7]. In Ireland, the number of SCD patients has also grown exponentially over the past two decades: from only 20 paediatric patients in 2000 [8] to over 500 adult and paediatric patients in 2020, a large proportion of whom are AYA. These first-generation Irish AYA SCD patients represent a dynamic patient population in Irish healthcare and present a unique view-point to inform global SCD research.

Significant data has accumulated supporting the safe, efficacious and cost-effective use of hydroxyurea in children and adults with SCD [9, 10] with important beneficial effects on quality of life [11–13]. Despite this evidence, however, underutilization of hydroxyurea in SCD remains a challenge and a number of factors have been identified, including provider, patient and system-related barriers [14, 15]. The most commonly identified patient barriers to adherence include forgetfulness, fear of side effects and access barriers [2, 3, 16–19]. Medication non-adherence among adolescents and young adults (AYA) with chronic illnesses, including SCD is particularly challenging [20]. However, there are a limited number of studies exploring the barriers to hydroxyurea adherence and this has never been investigated in an Irish context. Importantly, low hydroxyurea adherence rates are associated with worse HRQOL [3, 21] while improved adherence is associated with lower healthcare costs [22]. Therefore, understanding and overcoming the barriers to hydroxyurea adherence in AYA could substantially improve HRQOL and other health outcomes.

Recently, there is increased interest in the development of eHealth and technology-based platforms to improve patient care in chronic conditions [23, 24]. Indeed, there is promising evidence supporting the feasibility and efficacy of digital interventions for behaviour change, especially the use of smartphone applications in AYA [25–29]. Specifically, in the context of SCD, improved adherence to medication in children and adolescents using text-messaging reminders [30] and smartphone-based directly observed therapy has been demonstrated [31, 32]. Widespread availability and use of technology particularly by AYA present a unique opportunity for development of tools to promote medication adherence in this cohort.

The objectives of this study were (1) to examine hydroxyurea adherence rates and identify barriers to adherence such as recall, understanding and access barriers, (2) to explore HRQOL domains and explore the relationship between adherence barriers and HRQOL scores and (3) to identify smartphone app preferences for improving compliance in an Irish AYA SCD cohort. We hypothesized that recall barriers (forgetfulness) would play a prominent feature in the barriers to adherence faced by AYA and that participants with increased adherence barriers would report worse HRQOL outcomes.

## Methods

### Participant enrolment

Following local ethical approval, a cross-sectional study was performed using convenience sampling methodology at the National paediatric and adult SCD centres at Children’s Health Ireland (CHI), Crumlin and St James’s Hospital (SJH), Dublin, respectively. Patients were eligible if they were AYA (12–35 years) with SCD on hydroxyurea for 1 year or more prior to the study. In order to minimise selection bias, participants were recruited from a range of clinical settings, including the hydroxyurea clinic, transition clinic, haematology day ward and inpatient wards. The survey was entirely anonymous, with participants asked to specify only their age and gender.

## Study measures and survey design

Our survey was self-administered using pencil/pen and paper and comprised of 24 multiple choice or yes/no questions and a visual analogue scale (VAS) to assess adherence to hydroxyurea over the past month. Questions were inspired by the modified Morisky adherence scale 8 items (©MMAS-8) and Brief Medication Questionnaire (BMQ) to assess adherence rates and barriers, with some adapted elements of the Patient Reported Outcomes Measurement Information System (PROMIS ©) used to investigate HRQOL outcomes. All of these tools have been previously used in the context of AYA with SCD [2, 3, 33]. Questions pertaining to smartphone app preferences were based on available evidence in the literature related to app-based interventions and medication adherence in SCD [23, 34]. The final survey content and questions were reflective of multidisciplinary input from experts in the field of SCD including paediatric and adult Haematology, Nursing and Psychology. All self-reported adherence measures were adapted to reflect SCD and hydroxyurea and age-appropriate wording for AYA was used to minimize respondent burden.

## Statistical analysis

Descriptive statistics were reported in frequencies and percentages for categorical data. A sub-analysis was performed to assess whether age or presence of barriers to adherence influenced adherence rates, HRQOL parameters or preference of proposed smartphone app features. Participants were dichotomized by age group into adolescents (age 12–17 years) and young adults (age 18–35 years). The presence of recall barriers was defined if participants answered ‘yes’ to the question: ‘Do you sometimes forget to take your Hydroxyurea?’ and access barriers were present if respondents ‘sometimes’ or ‘always’ report finding it ‘difficult to take Hydroxyurea because my prescription runs out’. To assess statistical differences between the subgroups, data were analysed using the Mann-Whitney *U* test. A *P *value < 0.05 was considered statistically significant. Statistical analysis was conducted using GraphPad Prism 8.3 (GraphPad Software Inc., San Diego, CA).

## Results

### Characteristics of participants and overall adherence to hydroxyurea

Sixty-three participants were approached and 100% responded to the survey, 63% female and 37% male with a median and mean age of 17 (IQR 17–21) and 19.5 years, respectively. All participants were of African ethnicity and had homozygous SCD (HbSS). The average monthly self-reported adherence to hydroxyurea was 76% using a visual analogue scale (VAS). Overall, 73% of respondents feel hydroxyurea works either ‘very well’ (38%) or ‘reasonably well’ (35%) for them. Participants with high self-reported hydroxyurea adherence (> 80% according to VAS) perceived more beneficial effects of their medication (hydroxyurea ‘works very well for me’) compared with low (≤ 80%) adherence (hydroxyurea ‘works reasonably well’ or ‘I don’t know’) (*p* = 0.06). No statistically significant differences were observed among parameters between male and female respondents or between institutions.

## Adherence barriers and HRQOL

Recall barriers (forgetting to take hydroxyurea) were apparent in 62% of respondents, with 67% requiring prompts from family members to take their medication (Table 1). Twenty-one percent of participants omit hydroxyurea for reasons other than forgetting, while 11% discontinue their medication when they feel their SCD is under control and only 6% due to adverse effects (Table 1). Recall barriers/forgetfulness was the most common barrier to self-reported hydroxyurea adherence (62%) while access barriers (inability to obtain prescription/medication runs out) were evident in 33% of respondents (Table 1). Participants perceived minimal knowledge barriers with 97% reporting that doctors/nurses had clearly explained the reasons to take hydroxyurea and 92% self-reported a good understanding of their medication (Table 1).

Thirty-five percent of respondents worry about others knowing they take hydroxyurea while 54% worry about the long-term effects of the medication (Table 1). Eight-one percent reported that anxiety about health interferes with their lives (Table 1). Only 37% of respondents felt they were always physically able to do the activities they most enjoy while 51% experience disruption to school/college/work due to pain and 69% due to difficulty concentrating (Table 1).

## Participants interest in smartphone app and relevant features

Interest in a smartphone app to improve medication adherence was expressed by 87% (Table 1). While participants expressed interest in most of the proposed app features, the most popular features included: daily medication reminders (73%), adherence progress tracking (77%) and facts about SCD (72%) (Table 1). Discussion forums, sharing adherence data with doctors and rewards/incentives for using the app were less appealing (55%, 48% and 43% respectively) (Table 1). There was no statistically significant difference between app feature preferences between adolescents (12–17 years) and young adults (18–35 years) except for interest in a discussion forum, which was more preferable in the young adult group (*p *= 0.01) (Table 2).

## Subgroup analysis: age

Adolescents (12–17 years) comprised the majority (54%, *n* = 34) and young adults (18–35 years) represented 46% (*n* = 29) of the cohort. Overall, there was no difference in recall barriers/forgetfulness or monthly medication adherence using a visual analogue scale between these age groups. However, medication omissions for reasons other than forgetting were more frequent in the young adult group compared with adolescents (38% versus 15%, respectively, *p* = 0.05) and young adults were more likely to cut back on hydroxyurea due to ‘feeling worse’ when taking it (14% versus 0%, *p* = 0.04) (Table 2). Young adults were also more likely to report access barriers than adolescents (*p* = 0.04) but family prompting was less frequent in this subgroup (46% versus 82%, *p* < 0.001) (Table 2).

Young adults were more likely than adolescents to worry about the long-term effects of hydroxyurea (*p* = 0.02) and were also more likely to experience interference in their daily lives due to health worries (*p* = 0.005) (Table 2). Furthermore, young adults were significantly more likely to find their treatment plan bothersome compared with adolescents (*p* < 0.0001) (Table 2). Other than parameters related to anxiety, there was no significant difference observed between the groups in other HRQOL parameters including ability to perform physical activity, pain or fatigue interference or difficulty concentrating.

## Subgroup analysis: recall and access barriers and impact on adherence

Participants with recall barriers (62%) were more likely to report low hydroxyurea adherence over the past month using a visual analogue scale (median 80% versus 95% adherence, *p* < 0.0001) (Table 3). Additionally, significantly reduced physical ability was apparent in those with recall barriers compared to those without (*p* = 0.04) (Table 3). Participants with recall barriers were more likely to report difficulty remembering to take hydroxyurea at weekends, whereas those without such barriers were more likely to perceive ‘no difference’ between weekdays and weekends (*p* = 0.005) (Table 3). Similarly, those with recall barriers reported more difficulty remembering to take their medication at the same time every day compared to those who did not (*p* = 0.003) (Table 3). Overall, interest in a smartphone app was higher in those with recall barriers (*p* = 0.02) and interest in the medication reminder feature was also significantly higher (*p* = 0.003) compared with those without recall barriers (Table 3). Other than reduction in physical ability, there were no differences in HRQOL outcomes, anxiety or perceived benefits of hydroxyurea therapy between those with/without recall barriers.

Access barriers (difficulty in obtaining prescriptions) were reported in 33% of respondents, who were more likely to be older (median age 16 versus 19 years, *p* = 0.04) but there was no statistically significant difference between these groups across other adherence or HRQOL parameters. Average monthly hydroxyurea adherence rates according to visual analogue scale were 70% and 83% in those with and without access barriers, respectively; however, this difference was not statistically significant (*p* = 0.10).

## Discussion

With recent National Institute of Health (NIH) and British Society of Haematology (BSH) guidelines recommending the use of hydroxyurea in SCD patients from 9 months of age [35, 36], optimizing adherence across the lifespan is more essential than ever and understanding barriers to adherence may provide valuable and actionable insights. Our study contributes to the emerging literature on hydroxyurea adherence and is the first to examine adherence, HRQOL and attitudes towards technology-based platforms in an Irish context. The number of SCD patients in Ireland has expanded significantly over the past 20 years, reflecting the changing face of the Irish healthcare system. This dynamic patient population therefore represents a unique perspective  to inform international SCD research.

Forgetfulness was the most common barrier to hydroxyurea adherence with access barriers being less prevalent, findings which are consistent with other studies [3, 37, 38]. Although knowledge gaps and lack of information have been previously reported as barriers to adherence in SCD [19, 39, 40], these issues were less apparent in our cohort with 92% self-reporting a good understanding of their medication.

Despite this, levels of hydroxyurea- and overall health-related anxiety were high. Importantly, anxiety levels were significantly higher among young adults than adolescents. While elevated anxiety levels have been identified in children and adolescents with other chronic health conditions such as type 1 diabetes and haemophilia [41, 42], few studies have evaluated anxiety in AYA with SCD. Our findings suggest that health and medication-related anxiety is common and healthcare providers should be cognisant of anxiety among adolescents and especially young adults with SCD, which may simultaneously impact upon both quality of life and medication adherence.

Although we did not observe a difference in adherence between adolescents and young adults in this study, increased age and negative perceptions of medication have previously been identified as risk factors for non-adherence and worse HRQOL outcomes in SCD [2, 22]. The finding that young adults were significantly less likely to receive family prompting and are more likely to omit their medication deliberately (not due to forgetting) or due to perceived side effects than adolescents is reflective of patients’ increased independence as they grow older and exert more autonomy over their healthcare decisions. These data highlight the importance of self-management skill-building in AYA with SCD to promote hydroxyurea adherence in adulthood and later life.

Previous studies have shown that patients with higher hydroxyurea adherence rates have better HRQOL [3, 11, 12]. Consistent with these findings, we observed that participants with recall barriers had a significantly reduced hydroxyurea adherence using a VAS. Moreover, respondents with recall barriers were less physically able to do the activities they most enjoy. Due to the cross-sectional nature of this study, directionality of any cause-and-effect relationship between recall barriers and HRQOL outcomes such as physical ability cannot be determined. Additionally, in AYA with SCD, deficits in cognitive and executive functioning may be present which could reflect disease severity and impact HRQOL as well as contributing to recall barriers. Although we did not detect a statistically significant difference for other HRQOL domains (e.g. pain, fatigue and poor concentration), this could be related to our small sample size and relatively low statistical power.

Evidence from a recent systematic review of interventions to improve medication adherence in general including eHealth technologies suggest that smartphone applications have the potential to improve medication adherence in SCD [23, 43]. The majority of our cohort expressed interest in a potential app to improve hydroxyurea adherence. Interestingly, patients with recall barriers were significantly more likely to express an interest in the app, specifically in the daily reminder feature, than those without. This suggests that poor recall does not necessarily indicate a lack of patient engagement or willingness to take medication if appropriate prompting and support systems are available. Discussion forums were significantly more popular among young adults than adolescents, again reflective of the increased independence of the former group.

Our study has some limitations that warrant discussion. Firstly, this was a cross-sectional study using convenience sampling and the relatively small sample size may have constrained our ability to find statistically significant relationships between some hydroxyurea adherence barriers and HRQOL scores. Additionally, patients with poor attendance who did not get an opportunity to complete the survey may have provided further insight. Nevertheless, the fact that the study was carried out at two separate hospitals which are the largest and only tertiary SCD referral centres in Ireland adds strength to the findings and generalisability of the study. Furthermore, although absolute numbers of respondents are relatively low (*n* = 63), this sample represents over 10% of the Irish SCD population. Secondly, we evaluated hydroxyurea adherence using self-reported measures and this method may over-estimate adherence [16, 17, 44]. Supplementation of self-reported data with objective measures such as pill-counting or surrogate laboratory markers of adherence (e.g. haemoglobin F percentage) has been used in other studies [3, 45, 46]; however, this was not possible here due to the anonymous nature of our survey. Information pertaining to indications for treatment, current and maximum tolerated dose of hydroxyurea were also unavailable for the same reason. Finally, we did not explore details in relation to technology access or socioeconomic factors which may influence both adherence barriers and access to technology. However, studies in the USA indicate that the majority of AYA SCD patients have access to smartphones, findings which are likely generalizable to other developed countries such as Ireland [34, 47].

Future developments of any eHealth interventions including smartphone applications should include patient input from the outset of intervention design to ensure both long and short term engagement. Additional consideration must be given to cost analysis of any eHealth intervention going forward as there is a paucity of economic data to support the use of eHealth interventions to date [48, 49].

## Conclusions

Our findings enhance our understanding of SCD patient perceptions of and adherence to hydroxyurea and is the first to investigate this in an Irish context. While medication adherence among Irish AYA SCD patients appears relatively high, recall barriers are the most common impediment to adherence and are associated with worse HRQOL. A potential smartphone app to improve adherence was welcomed and the strong preference for daily medication reminders reflects the prevalence of recall barriers in this cohort.

Representing over 10% of the Irish SCD population, our study provides novel and valuable insights into medication adherence and HRQOL domains in AYA. Importantly, data regarding preferred app features may inform future technology-based interventions to improve medication adherence for AYA with SCD as well as other chronic health conditions.

**Table 1:** Survey results total AYA cohort (*n* = 63)

Adherence and understanding	Yes %	No %	
Sometimes forget to take hydroxyurea	62	38	
Need for parents/family reminders to take hydroxyurea	67	33	
Forget to bring medication when travelling or leaving home	16	84	
Stopped taking hydroxyurea for reasons other than forgetting	26	74	
Stopped taking hydroxyurea as prescription/medication ran out	33	67	
Cut back/stopped taking hydroxyurea due to adverse effects	6	94	
Stopped taking hydroxyurea when SCD is under control	11	89	
I have a good understanding of why I take hydroxyurea	92	8	
Doctors/nurses clearly explained the reasons to take hydroxyurea	97	3	
Hydroxyurea protects my health from becoming worse	94	6	

Anxiety and HRQOL domains	Always %	Sometimes %	Never %
Trouble doing things at school/college/work due to pain	10	41	49
Difficulty keeping up at school/college/work due to fatigue	13	58	29
Physically able to do the activities I enjoy most	37	56	7
Difficulty concentrating at home or at school/college/work	15	54	31
Worrying about my health interferes with my life	27	54	19
Worry about other people knowing I take hydroxyurea	8	27	65
Worry about the long-term effects of hydroxyurea	10	44	46

Smartphone application preferences	Yes %	No %	
Interest in a smartphone app to improve hydroxyurea adherence	87	13	
Interest in daily medication reminders	73	27	
Interest in a discussion forum to talk to others about SCD	55	45	
Interest in receive facts about SCD and its treatment via the app	72	28	
Interest in progress tracking for medication adherence	77	23	
Interest in sharing data from the app with doctors	48	52	
Interest in receiving incentives for daily app usage	43	57

**Table 2:** Subgroup analysis by age: adolescents (12–17 years) or young adults (age ≥ 18 years)

	Adolescents {12–17 years *n *= 34}			Young adults (> 18 years *n* = 29)			
Subgroup analysis: age	Yes%	No%		Yes%	No%	*p* value	
Omitted other than forgetting	15	85		38	62	*p* = 0.048	
Cut back due to adverse effects	0	100		14	86	*p* = 0.042	
Forgetfulness/recall barriers	62	38		62	38	*p* > 0.99	
Interest in smartphone app	84	16		90	10	*p* = 0.71	
Interest in discussion forum	39	61		72	28	*p* = 0.01	
% Monthly adherence (VAS)		% Monthly adherence (VAS)		74 (median 85)		*p* = 0.46	
	Always %	Sometimes %	Never %	Always %	Sometimes %	Never %	*p* value
Family prompting	29	53	18	0	46	54	*p* = 0.003
Worry about long term effects	3	38	59	17	52	31	*p* = 0.02
Bothered by treatment plan	9	50	41	38	59	3	*p* < 0.001
Worry interferes with daily life	18	50	32	38	59	3	*p* = 0.005

**Table 3:** Subgroup analysis by presence or absence of recall barriers (forgetfulness)

Subgroup analysis: recall barriers	Recall barriers present			Recall barriers absent			
	Yes%	No%		Yes%	No%	*p* value	
Interest in smartphone app	95	5		73	27	*p* = 0.02	
Interest in daily reminder feature	87	13		50	50	*p* = 0.003	
% Monthly adherence (VAS)		70 (median 80)		93 (median 95)		*p* < 0.001	
	Always %	Sometimes %	Never %	Always %	Sometimes %	Never %	N ever %
Phy sically able to do recreational activities	28	59	13	50	50		*p* = 0.04
Difficulty taking at the same time each day	23	56	21		33	59	*p* = 0.03
Harder to take during the week or weekends	Week 20%	Weekend 31%	Equal 49%	Week 5%	Weekend 86%	Equal 86%	*p* = 0.005

## Data Availability

The data that support the findings of this study are available from the corresponding author upon reasonable request.
